# Roles of Mucosal Immunity against *Mycobacterium tuberculosis* Infection

**DOI:** 10.1155/2012/791728

**Published:** 2012-11-01

**Authors:** Wu Li, Guangcun Deng, Min Li, Xiaoming Liu, Yujiong Wang

**Affiliations:** ^1^Key Laboratory of Ministry of Education for Conservation and Utilization of Special Biological Resources in the Western, Ningxia University, Yinchuan, Ningxia 750021, China; ^2^College of Life Science, Ningxia University, 539 W. Helanshan Road, Xixia District, Yinchuan, Ningxia 750021, China

## Abstract

*Mycobacterium tuberculosis* (Mtb), the causative agent of tuberculosis (TB), is one of the world's leading infectious causes of morbidity and mortality. As a mucosal-transmitted pathogen, Mtb infects humans and animals mainly through the mucosal tissue of the respiratory tract. Apart from providing a physical barrier against the invasion of pathogen, the major function of the respiratory mucosa may be to serve as the inductive sites to initiate mucosal immune responses and sequentially provide the first line of defense for the host to defend against this pathogen. A large body of studies in the animals and humans have demonstrated that the mucosal immune system, rather than the systemic immune system, plays fundamental roles in the host's defense against Mtb infection. Therefore, the development of new vaccines and novel delivery routes capable of directly inducing respiratory mucosal immunity is emphasized for achieving enhanced protection from Mtb infection. In this paper, we outline the current state of knowledge regarding the mucosal immunity against Mtb infection, including the development of TB vaccines, and respiratory delivery routes to enhance mucosal immunity are discussed.

## 1. Introduction 

Tuberculosis (TB) is one of the world's leading infectious disease with approximately two million deaths and eight million new cases annually. It is also a severe pulmonary disease and a public health burden caused by the infection of *Mycobacterium tuberculosis* (Mtb) [1]. Mtb is a facultative intracellular bacterium capable of surviving and persisting in host mononuclear cells where it is able to escape the elimination through numerous mechanisms [2]. The capacity of Mtb to survive within a host cell for decades without replicating may be partially due to the fact that it is a metabolically, fastidious, acid-fast bacillus that grows very slowly, as well as its ability to inhibit phagosomal maturation by preventing phagosome-lysosome fusion and acidification of the phagosome [3]. Transcriptomic analysis has revealed that Mtb was able to gain its abilities to evade the host immune surveillance, adopted its specialized intracellular niche, and resisted various agents and antibiotic drugs, by expressing various genes against the host immune responses [4]. The ability of Mtb to evade the host immune surveillance and establish a latent metabolic state in the host causes the difficulty to eradicate tuberculosis, even though most of patients infected with Mtb could be cured with appropriate therapy. In addition, the reactivation of Mtb at a latent state in immunocompromised patients and the emergency of the multidrug-resistant Mtb strains and a coinfection with HIV have also increased the difficulty to prevent this disease [5]. To date, vaccination remains one of the most effective approaches for controlling TB worldwide. 

It is well known that the mucosa is the largest immune organ in the body, and it is generally believed that almost all infectious diseases are initiated at mucosal surface [6]. The respiratory tract is the natural route for Mtb infection, where Mtb infects the individual mainly through the mucosal tissue of the respiratory tract after inhalation of mycobacteria-containing droplets from the external environment. Normally, the pathogen (Mtb) infection could be eliminated by the host's immune system, but it is desirable to induce immunity prior to the infection by means of vaccination in most of the cases. In order to effectively prevent Mtb infection, the approach of mucosal immunization has recently received increasing attention in the field of tuberculosis vaccination owing to its potency in inducing mucosa-associated protection from mucosal infectious diseases [7–9]. Several lines of evidence have suggested that mucosal immunity can provide unique advantages for protection against mycobacterial infection, by which the immune cells, such as macrophages, dendritic cells, and leukocytes recognize the pathogen associated molecular patterns (PAMPs), and sequentially activate the antimycobacterial immune responses including the activation of specific T-cell and antibody synthesis [10–13]. 

In general, the mucosal protection includes the physical and chemical mucosal barriers, and a wide range of immune components for the recognition of invading pathogen by different cell types, the secretions of antimicrobial peptides, and factors of immune mediator/effector. This paper aims to summarize our current understanding of the mucosal immunity against Mtb infection, and some ongoing approaches of developing mucosal TB vaccines to enhance the mucosal immunity.

## 2. The Mucosal System in the Respiratory Tract

The immune system is composed of two primary compartments: the mucosal immune system and the systemic immune system. The mucosal immune system functions as the first line of defense against pathogens and is composed of inductive sites and effector sites. The inductive sites are responsible for antigen uptake and priming of naïve T and B cells that then migrate to other mucosal effector sites, while the effector sites are the mucosa where secretory IgA (sIgA) is produced and mucosal immunity is initiated [14]. In the mucosal surface, the epithelial cells line to establish a barrier and perform its “barrier functions,” and the immune cells play vital roles in host defense against infection of pathogens by migrating to the lamina propria of respiratory tract and other sites after they are primed. 

Mucosal surfaces are classically defined as the body's mucus-covered surfaces and include surfaces of the respiratory, gastrointestinal, and urogenital tracts as well as the exposed cornea/conjunctiva [15]. They are constantly in contact with external environments to perform physiological functions including nutrient transport, ion and water homeostasis, and respiration. The surface area of human adult mucosa is about 400 m^2^, and almost 80% of the total immune cell population is present at mucosal sites, with over 90% of human infections at the body's mucosal borders. Additionally, most of mucosal surfaces contain specialized mucosa-associated lymphoid tissues (MALT); the MALT consisted of gastrointestinal-associated lymphoid tissue, bronchial and nasal-associated lymphoid tissue (B/NALT), and conjunctiva- and urogenital-associated lymphoid tissue [15], all of which are necessary for antigen sampling and induction of mucosal immune responses [16]. The attachment of bacteria to mucosal surfaces is the first step in the pathogenesis of most infectious diseases; equally important, it is the first-line defense of mucosa against invading microbial pathogens. Thus, the mucosal immune system has been suggested to be the port of entry for many pathogens and is one of the most important immune organs against Mtb infection in the body. 

### 2.1. The Role of Mucosal Epithelial Cells in Mucosal Immunity against Mtb

In humans and animals, the mucosal surface is lined by epithelial cells and mucus-secreting cells that form tight barriers separating the external environments from the internal compartments. It is an important interface bridging the host cells with the environments. Since mucosal epithelial cells are constantly exposed to external environments, they are vulnerable to microbial attacks and play active roles in regulating mucosal immune responses by locally adapting of microbial recognition, maintaining of immune homeostasis, and modulating of antigen-presenting cells and adaptive immune responses, during the interaction of host and external pathogens (see review [17]). In addition, the interaction of epithelial cells with Mtb is an important step for Mtb to entry into the host body. Teitelbaum et al. found that epithelial M cells play important roles in this process [18]. Furthermore, a microbial attack may damage the mucosal layer; the mucosal epithelial cells, however, are able to rapidly restore the integrity of mucosal epithelium by initiating a programmed series of interdependent responses after the injury [19].

The mucosal epithelial cells play a prominent role in protecting the host from the invasion of pathogens through secreting many kinds of antimicrobial substances into the mucosal fluid (e.g., mucins, defensins, lysozyme, nitric oxide, and others), among which the production of sIgA is one of the important activities of mucosal epithelial cells. These defensive compounds in the mucosal surfaces form a physical barrier and have direct antimicrobial activity. Mucin glycoproteins are secreted in large quantities by mucosal epithelia, and they play a central role in accommodating the resident commensal flora and limiting infectious disease [20]. Defensins have an activity against a broad range of pathogens, which are a family of evolutionarily related vertebrate antimicrobial peptides with characteristics of *β*-sheet-rich folds and frame work of six disulphide-linked cysteines [21]. Defensins can act on the mycobacterial cell envelope and disrupt the membrane architecture to directly kill bacillus [22]. Such an antimycobacterial activity has also been proved in both mouse models [23] and humans [24]. Nitric oxide and other reactive nitrogen intermediates (RNI) produced by alveolar macrophages also aid the host's defense against the infection of Mtb (see review [25]). Furthermore, the mucosal epithelial cells can also secrete a series of proinflammatory cytokines against Mtb (see Section 4). These proinflammatory factors can eradicate organisms and infected cells by recruiting and activating phagocytic cells [26–28]. The mucosal epithelial cells also produce other compounds important for host against the invasion of Mtb. For example, surfactant protein A and surfactant protein D, which are produced by pulmonary epithelial cells, play important roles in the innate immunity by influencing the ability of pathogens to be taken up by host cells and/or cleared by host defense mechanisms [29]. Together with the family of Toll-like receptors, the surfactant proteins also play a key role in the recognition and binding of the pathogen to epithelial cells during Mtb infection (see review [30]).

### 2.2. The Role of sIgA in Mucosal Immunity against Mtb

It is generally accepted that cell-mediated immunity (CMI) plays a pivotal role in the immune response against Mtb infection. However, the protective role of Mtb-specific humoral immunity remains controversial. Recently, several lines of evidence showed a protective role of antibodies against Mtb [31–33], where sIgA is the most abundantly produced natural antibody isotype in mucosal tissue. sIgA has been found to play important roles in the exclusion of different antigens from mucosal surfaces. It serves as a first line of defense against pathogens in mucosal areas by agglutinating potential invaders and facilitating their clearance by peristaltic and mucociliary movements [34]. In addition, sIgA is the most characteristic component of the mucosal immunity [35].

sIgA can serve as a carrier of antigens; it also has the potential functions in the inhibition of bacterial adherence, neutralization of toxin and virus, and prevention of antigen uptake by epithelial cells, in part through a mechanism of binding and intercepting the invasion of pathogens and/or neutralizing their toxic products, during the transcytosis or in the mucosal fluids [36–39]. These imply that sIgA in mucosal secretions may play an important role in the host's early defense against invading pathogens in respiratory tract [40]. The above notion is supported by a previous human study in which the mucosal BCG vaccination could induce significant increases of sIgA [41]. This finding was also attested in a recent study using a mouse model; mice vaccinated with Ag85A-Esat-6-IL-21 DNA vaccine showed an improved level of sIgA in the bronchoalveolar lavage (BAL) [42]. Moreover, the sIgA could prevent the adsorption of pathogens at the mucosal epithelium [37–39]; the initial infection of Mtb could therefore be blocked at the mucosal surface by sIgA. Such finding was supported by a study in a murine model in which the entrance of mycobacterial bacilli into the lungs was blocked by sIgA [43]. 

## 3. Mucosal Immune Effector Cells against Mtb Infection 

The immune cell types in MALT include a variety of phenotypical and functional distinct T-cell, B-cell, and accessory cell subpopulations, by which the MALT plays a central role in regulating mucosal immunity. It has been well established that the cell-mediated immune responses (CMIRs) are the primary defense against intracellular pathogens such as Mtb infection. The functional “effectors” of these responses are various immune cells. In response to Mtb infection, the CD4^+^ and CD8^+^ subsets of T-cell population, as well as alveolar macrophages and dendritic cells (DCs), are the major immune effector cell types, which have been well documented by others; here we will only focus on the functions displayed by these immune cells in the mucosal microenvironment.

### 3.1. CD4^+^, CD8^+^, and Other T-Cell Subsets

As an intracellular pathogen, Mtb resides within the vacuole of host macrophages; the CD4^+^ T cells and CD8^+^ T cells are of primary importance in the host's protection against Mtb. CD4^+^ T cells are involved in primary resistance to Mtb by producing IFN-*γ* and other cytokines to activate macrophages, of which it is critical in the controlling and eliminating of Mtb [25]. In response to Mtb infection, the host initiates the CD4^+^ T-cell responses in the mediastinal lymph node (MLN) [44] and lung-draining lymph node (LN) [45, 46]. The critical role of CD4^+^ T cells in defending Mtb infection became evident during epidemiological studies of HIV-1 infection [47]. When mice were challenged with Mtb via an aerosol method, the CD4^+^ T cells in the lungs were highly activated during the acute phase of infection [48]. Recent study by Khader and coworkers also found that when mice were vaccinated, antigen-specific CD4^+^ T cells can be induced in the lungs of vaccinated mice [49]. Additionally, mice vaccinated with a recombinant adenovirus-basis TB vaccine delivered by a mucosal route exhibited an increasing number of Ag-specific CD4^+^ and CD8^+^ T cells, while parenteral vaccination intramuscular injection (i.m.) failed to elicit airway luminal T cells and protect the lung from Mtb infection in mice [50]. These studies suggested the important roles of CD4^+^ T cells in the mucosal immunity against Mtb. Interestingly, increasing evidence suggests that the effector CD4^+^ T-cell responses to Mtb are apparently delayed [45, 46, 51]. Although the reason for the delay is not completely understood, the delay was suggested to be likely to allow the bacteria sufficient time to establish persistent infection [44].

Other CD4^+^ T lineages cells, such as Th17 lineage cells, CD4 TEM cells, invariant T cells, and regulatory T cells, have also been demonstrated to be important for mucosal immunity against Mtb. For example, Th17 cells, which are distinct lineage of CD4^+^ T cells [52], play crucial roles in mucosal immune responses to major respiratory pathogens, and they are capable of regulating the production of antimicrobial proteins in mucosal epithelium and clearing various pathogens [53, 54]. In addition, regulatory T cells are important to maintain peripheral tolerance and homeostasis at mucosal surface [55]. 

CD8^+^ T cells are another subset of T cells necessary for the clearance of intracellular pathogens at mucosal sites. There seem to be three primary effector functions for CD8^+^ T cells in tuberculosis as compared to CD4^+^ T cells; these include lysis of infected cells in the mucosal surfaces (e.g., macrophages and DCs), direct killing of the intracellular bacteria, and production of IFN-*γ* cytokines. Similar to CD4^+^ cells, CD8^+^ cells are also important sources of IFN-*γ* against Mtb infection, even if the production is less relative to CD4^+^ T cells. The importance of CD8^+^ cells was also evidenced by the appearance of Mtb antigen (Ag)-specific CD8^+^ cells in the airway lumen at the time of Mtb infection [50, 56, 57]. Murine study suggested that intranasal (i.n.) delivery of low dose of soluble Mtb Ags was able to recruit and retain Ag-specific CD8^+^ cells in the airway lumen over time, while the Ags delivered via an i.m. route failed to induce the Ag-specific CD8^+^ cells [58]. Intranasal vaccination of recombinant adenoviral TB vaccines showed the accumulation and retention of antigen-specific CD8^+^ and CD4^+^ T cells in the airway lumen of mice [59]. Recent study by Mu and coworkers also found that CD4^+^ T-cell-depleted mice intranasally vaccinated with adenovirus vector expressing Mtb antigen Ag85A led to suboptimal generation of Ag-specific CD8^+^ T cells in the lung and spleen at the early time following the immunization [60]. 

Except CD4^+^ T cells and CD8^+^ T cells, other subsets of T cells such as *αβ* T cells and *γδ* T cells also have been found to play important roles in the mucosal immunity against Mtb. For instance, *γδ* T cells are abundant on mucosal and epithelial surfaces; they are capable of lysing infected macrophages and containing mycobacterial growth [61, 62]. By using a pulmonary murine model of Mtb, Lockhart and coworkers found that the major producers of IL-17 in T cells isolated from the lungs of infected mice were *γδ* T cells; this suggested that *γδ* T cells are more potent producers of IL-17 during the early immune response at mucosal sites following the infections [63]. 

### 3.2. Mucosal Dendritic Cells

There are two types of dendritic cells (DCs), tissue DCs (refer to those that reside in the peripheral tissues such as mucosa, skin, and internal organs) and blood DCs [64]. Mucosal DCs, especially the airway mucosal DCs, are key effectors in response to Mtb infection. In tuberculosis, DCs are involved in the induction of antimycobacterial T-cell immune response; Mtb-infected DCs have the ability to produce interferon [65] and phagocytose the Mtb in murine model [66, 67]. In the airway mucosa, DCs are present in the epithelium and underlying lamina propria, as well as in the lung parenchyma and alveolar spaces of the lower respiratory tract [68]. They are the first responders to infection at epithelial surfaces of mucosal tissues, in which DCs are distributed throughout intra and subepithelial sites. Mucosal DCs are able to recognize pathogens through engagement of pattern recognition receptors (PRRs) [69]. Upon an appropriate stimulation, DCs undergo further maturation and migrate to secondary lymphoid tissues where they present Ags to T cell. The activated effector T cells then migrate back to the infection sites to produce cytokines, activate macrophages, and lyse target cells for eliminating the pathogens. Thus, DCs are unique antigen-presenting cells (APCs) with a capacity to stimulate naïve T cells. In response to Mtb, the invasion of this pathogen may stimulate DCs to initiate immune responses, but they also may impair the function of DCs function to provide a mean of immune evasion for Mtb [70]. The constituents of mycobacteria possess a capacity to induce the activation and maturation of DCs [71–73]. However, exposure of attenuated or virulent Mtb could cause the death of human DCs [74]. 

### 3.3. Alveolar Macrophages

Macrophages, especially alveolar macrophages, are important immune cells against Mtb infection. It is generally accepted that alveolar macrophages are one of the first cell types that encounter Mtb in the lungs. They are a primary host cell type for Mtb to live in and the first-line of defense in the lung against infection of these bacilli. Mtb could survive and grow in the activated macrophages [75, 76] or be killed by the host cells through a mechanism of producing reactive nitrogen intermediates [77, 78]. As a successful pathogen, virulent Mtb is capable of escaping the host's immune surveillance and living and replicating in the resting macrophages, partially by a mechanism of impairing macrophage plasma membrane repair [79, 80].

A large body of study has shown that autophagy plays a role in innate immune responses to intracellular bacterial infections. Murine studies have shown that the autophagy participated in the process of mycobacterial destruction in infected macrophage, although a rapid macrophage apoptosis could be induced after the infection [81]. These results suggested that the autophagy might contribute an important part for the host to defend against Mtb invasion [82–85]. 

Phagolysosome fusion is another antimycobacterial mechanism of macrophages, which is an evident mechanism for the mononuclear phagocyte lineage to inhibit and kill intracellular pathogens within cells [86]. Fusion of the lysosome with a phagosome-containing ingested bacterium is a primary mechanism by which macrophages kill a pathogen. In an activated macrophage, phagosomes in which the pathogens are enclosed fuse with lysosomes to form phagosomes and kill the pathogens. However, mycobacteria have evolved mechanisms to inhibit phagosome maturation, prevent it fusion with lysosomes, acidify, and expose bacteria to lysosomal hydrolases [87, 88]. Hence, the inhibition of autophagosome formation may be one of the most important mechanisms for Mtb to escape from host immune surveillance [89]. 

## 4. Cytokines in Mucosal Immunity against Mtb

Mtb infection usually results in the induction of large number of cytokines. These cytokines are key effectors in the host defense against Mtb (see review [25]), among which IFN-*γ*, TNF-*α*, IL-12, and granulocyte-macrophage colony-stimulating factor (GM-CSF) are of most importance in the mucosal immunity to Mtb. IFN-*γ* is a critical cytokine in protective immunity against Mtb. Except for CD4^+^, CD8^+^ T cells, and NK cells [90], pulmonary mucosal epithelial cells are another source of IFN-*γ* in response to Mtb infection [91]. IFN-*γ* can activate infected macrophages to directly inhibit the intracellular replication and growth of Mtb in the macrophages. IFN-*γ*-deficient mice challenged with a sublethal dose of Mtb showed a loss of cohesive structure of multiple foci of bacterial growth in the lung, suggesting a key role of IFN-*γ* in primary Mtb infection [92]. Another murine study demonstrated that mice immunized with Ag85A DNA vaccine through an oral route showed the expression of targeted antigen in the mucosal epithelial cells of the small intestine, striking increased levels of IFN-*γ* in the intraepithelial lymphocytes (IELs), as well as relevantly decreased bacterial loads, in the lungs in comparison with the control group [93]. Furthermore, the depletion of IFN-*γ* by antibodies could lead to increased number of Mtb in the lungs of mice, which strongly implied that IFN-*γ* might play critical roles in governing the outcome of latent infection [94]. Similar function of IFN-*γ* was found in human studies; individuals deficient in functional IFN-*γ* or IFN-*γ*-receptor exhibited an enhanced susceptibility to Mtb [95, 96]. *In vitro* study using human lung epithelial cells also showed an increased production of IFN-*γ* and IFN-*γ* receptors in A549 cell infected with Mtb, indicating that IFN-*γ* might play important roles in innate immunity against tuberculosis [91]. In addition, a direct evidence of defective macrophage activation in IFN-*γ* knockout mice showed that IFN-*γ* was responsible for activation of infected macrophages following Mtb challenge [97]. Apart from its function in activating macrophages to inhibit intracellular growth of Mtb, IFN-*γ* is also a critical regulator for APC by increasing MHC and costimulatory molecular expression [98]. Moreover, IFN-*γ* can be used as an index for the diagnosis of tuberculosis infection, which is able to avoid cross-reactivity with BCG immunization and nontuberculous mycobacterial infections [99].

TNF-*α* is another important immune mediator secreted by the activated macrophages and lung epithelial cells, which is capable of stimulating an acute phase reaction in response to Mtb infections. This mediator is able to play multiple roles in the host's immune response and pathogenesis of tuberculosis. By eliciting the production of TNF-*α*, Mtb may gain a capability to penetrate alveolar epithelium after infection. On the other hand, TNF-*α* is essential for the initiation of immune response against Mtb infection [114, 115], and for an effective granuloma formation. The formation of granuloma is important in mediating prolonged containment of mycobacteria, and it has been suggested to be the hallmark of containment of Mtb bacterial growth in the lung [116, 117]. Like IFN-*γ*, TNF-*α* is another important component for macrophage activation [117] and induction of apoptosis and necrosis of infected macrophages [118, 119]. In spite of its essential roles in the host's immunity to Mtb infection, overproduction of TNF-*α* in pulmonary TB may cause fevers, weakness, night sweats, necrosis, and progressive weight loss [120]. Experiments with recombinant BCG-expressing TNF-*α* demonstrated that high levels of TNF-*α* could cause destructive inflammation; the relative amount of TNF-*α* at the site of infection determined whether the cytokine acts as a protective or a destructive mediator [121].

In addition to IFN-*γ* and TNF-*α*, IL-12 is another cytokine with an immunoregulatory function that bridges the innate and adaptive immunities [122]. IL-12 is mainly produced by macrophages, which is essential for immunity against of Mtb infection. IL-12 p40^−/−^ mice, genetic modified mice lacking the gene for the IL-12 p40 subunit, have been found to be extremely susceptible to Mtb infection as compared with the C57BL/6 WT mice. It is thought that exogenous administration of IL-12 to mice was able to increase resistance to the infection and improve the granuloma formation, while a depletion of IL-12 with specific antibodies altered granuloma formation [115, 123, 124]. These studies indicated an important role of IL-12 in the formation of protective granulomas. However, IL-12 could not induce a protection against Mtb in the absence of IFN-*γ* in mice [115]. Other biological functions of IL-12 include its ability to increase the production of IFN-*γ* and activate naïve NK and T cells [123, 125]. 

GM-CSF is another important cytokine that contributes to the control of Mtb infection and the containment of Mtb growth, which is produced by many cell types including airway epithelial cells, macrophages, and type II alveolar epithelial cells [126]. The production of GM-CSF induced by rBCG is enabled to enhance antimycobacterial T-cell responses and could improve immune protection [127]. Mice with GM-CSF gene disruption were unable to contain the bacterial growth in the lungs, while the GM^+^ mice were able to limit bacterial growth to an extent. Unlike those seen in their wild-type littermates, GM-CSF disrupted mice and GM^+^ mice did not exhibit a detectable granuloma formation in the lungs where the GM-CSF was overexpressed; this indicated that GM-CSF might play a role in the granuloma formation [128]. In addition to its immunoregulatory function, GM-CSF is also an effective adjuvant and immune regulatory molecule for vaccine development against TB [129, 130]. 

## 5. Mucosal Adjuvants and Vaccine Candidates against Mtb 

The mucosal immunity has been suggested to play central roles in TB. However, most conventional vaccines are designed for delivery via systemic routes, which augment systemic immunity, but induce a lesser immune response at the mucosal site of pathogen entry. Increasing numbers of studies have documented that the respiratory mucosa provides a valuable target site for immunization against respiratory pathogens including Mtb. The Bacillus Calmette Guerin (BCG), the only licensed vaccine against TB, has been used for almost a century. However, BCG does not protect all age groups, and its protective efficacy in adults is highly varied from different trials with complications (0–80%) [131–133]. Additionally, the BCG-induced protection lasts no longer than fifteen years [134], and it neither prevents the establishment of latent tuberculosis infection, nor is suitable for use with immunocompromised patients. Therefore, novel and more effective vaccines and vaccination strategies are needed. 

Despite the great efforts that have been focused on producing an effective vaccine capable of inducing a cell-mediated immune response to kill the intracellular bacilli, such vaccine candidates and approaches have not induced a significantly better protection than BCG. It has been extensively demonstrated that mucosal immune response and mucosal antibodies (in particular the sIgA) are important in protecting the host from Mtb infection [36, 135]. The vaccines, adjuvants, and delivery strategies attempted to enhance respiratory mucosal immunity will help gain further attention in the research of TB vaccine (Table 1). 

A range of subunit antigens of Mtb have been tested as antimycobacterial vaccine candidates, such as the 6-kDa early secretory antigenic target (ESAT-6), 10-kDa culture filtrate protein (CFP-10), a 30 to 32 kDa family of three proteins (Ag85A, Ag85B, and Ag85C), MPT64, MPT83, Hsp65, and KATG of the bacilli; these subunit antigens were used either alone or in combination (Table 1). As a mucosal pathogen, a vaccine capable of efficiently inducing mucosal responses may offer a desirable protection against Mtb. Indeed, increasing evidence does suggest that vaccination via a mucosal route produce better protective effects relative to a conventional intramuscular or subcutaneous injection against mucosal infectious diseases [136]. Currently, great efforts have been devoted to improve the protective efficacy of TB vaccines. These efforts include genetical modification of BCG (rBCG), defining and producing protective subunit antigens or epitopes, and development of adjuvant, vehicles, and/or vectors for targeting mucosal surfaces, as well as delivery strategies for enhancing mucosal immunity. For instance, Ballester and coworkers recently developed a synthetic vaccine delivery platform with nanoparticles (NPs) loaded with the tuberculosis antigen Ag85B; such a formulated vaccine candidate was capable of inducing mucosal and systemic Th17 responses in mice [137]. A strategy of multistage vaccination, in which the Mtb antigens Ag85B and ESAT-6 were combined with the latency-associated protein Rv2660c, was able to control reactivation of Mtb and significantly lower the bacterial load [138].

Three criteria may be essential for determining a successful mucosal immunization strategy: effective delivery of antigen to the mucosal immune inductive site, the enhancement of mucosal immune responses by the use of mucosal immunomodulators (adjuvant), and the route of immunization [139]. It has long been recognized that most vaccines require the addition of adjuvant to enhance their immunogenicity, particularly in the mucosal immunization with soluble proteins or peptides, of which vaccination with such soluble immunogens without mucosal adjuvant may induce a state of antigen-specific immunological tolerances [140]. BCG itself is an effective adjuvant and has been used for the active immunotherapy of various cancers for many years [141]. Recently, whole cell lysate of Mtb was also used as a potent mucosal adjuvant [142]. In addition to conventional aluminum salts, oil-in-water emulsions [143], components of microbial origin (DNA motifs, lipid A, cholera enterotoxin (CT), and *Escherichia coli* heat-labile enterotoxin (LT)) [144, 145], emulsions and particles (immunostimulating complexes (ISCOMs), liposomes, PLGA, and saponins) [146, 147], Eurocine L3 [148], and cytokines are in use or under development for adjuvant and/or vaccine delivery vehicles. Notably, ISCOMs, CT, LT, Eurocine L3, and saponins are the most available mucosal adjuvants. For example, antigen encapsulated in biodegradable poly-L-lactide microspheres was capable of conferring an adjuvants effect [149], and the mucosal delivery of Mtb in microspheres enabled to induce robust cell-mediated responses in the lungs [106]. 

Apart from the immunogenicity and adjuvanticity, an appropriate antigen delivery route and system for vaccination is also critical for an effective immunization. Since the primary targeting site of Mtb is in respiratory mucosa, the respiratory mucosal immunization has received increasing attention recently. Mucosal vaccination via the respiratory tract such as intranasal delivery, displays many advantages over other routes including the subcutaneous immunization. The intranasal delivery approach is much easier and more flexible, and more importantly, mucosal delivery exhibited a capacity to trigger both mucosal and systemic immune responses [150]. Vaccination through an intranasal route was also superior to the subcutaneous route in the protection against pulmonary TB [151]. Additionally, it has been demonstrated that intranasal immunization of antigens with mucosal adjuvant could effectively induce the production of sIgA. The sIgA was thought to play an important role in the host's defense against mucosal pathogens including the Mtb [152, 153].

## 6. Concluding Remarks

Mtb is an airborne transmitted pathogen, and the immune responses, especially the mucosal immune response, play fundamental roles for the host to defend the primary and the containment of Mtb infection. Despite the fact that BCG has made a tremendous contribution to the control of Mtb infection, particularly in child population and newborns, there is not a consistent effective vaccine available for TB. Therefore, the development of novel, safer, and more effective vaccines and vaccination strategy capable of conferring a broad protection at the respiratory mucosa is required. Given the fact that more than one-third of the population is infected with Mtb, but only 10% of them develop active disease while >90% of all who contained infections remain dormant, suggests that the variability of immune responses may be attributed to the outcome of Mtb infection. There is growing evidence demonstrating that the mucosal immunity plays a central role in the host against Mtb infection; thus, a better understanding of the mucosal immunity will aid us to improve the diagnostic procedures and the development of efficient vaccines against TB. Nevertheless, there are surprising gaps in the knowledge of mucosal immunity against Mtb, which highlight the need for additional research to fully understand the mechanisms of mucosal immunity, especially mechanisms of immune evasion by Mtb and molecular pathogenesis of Mtb. 

## Figures and Tables

**Table 1:** Respiratory delivery of vaccine candidates against mycobacteria infection^+^.

Antigen	Delivery route and/or adjuvant	Tested species	Results or immune responses	Reference
Live rBCG	Delivered by intranasal (i.n.) route	Mice	Induce strong antibody responses	[100]
Live BCG	Delivered aerosol inhalation	Possum	Protection from *M. bovis* infection	[101, 102]
Live BCG	Delivered by i.n. route	Mice	Protection from H37Rv challenge	[103]
Live BCG	Delivered by i.n. route	Mice	Increase protective effect of BCG vaccine	[11]
Mtb cell wall MDP	Delivered by aerosol inhalation	Guinea pig	Activate alveolar macrophages	[104]
Killed BCG	Delivered in Eurocine L3 adjuvant *via* i.n. route	Mice	Induce high immune responses	[105]
ESAT-6 protein	Delivered by PLA microsphere *via *i.n. route	Mice	Induce specific immune responses	[106]
FbpA, HtpX	Intranasally delivered by an *E. coli* vector	Mice	Induce specific T-cell response and protection from Mtb challenge	[107]
Ag85A	Delivered with adenoviral and VSV vectors *via* i.n. route	Mice	Induce mucosal T-cell response	[108]
HSP65 (DNA)	Delivered by liposome *via* i.n. route	Mice	Induce strong cellular immune response	[109]
Ag85A, CFB10	Adenoviral vector *via* i.n. route	Mice	Induce specific immune response and protection from Mtb challenge	[33, 110, 111]
Ag85A	Delivered with Adenoviral and VSV vectors *via* i.n. route	Mice	Induce mucosal T-cell response	[108]
HSP65 (DNA)	Delivered by liposome *via* i.n. route	Mice	Induce strong cellular immune response	[109]
Ag85A, CFB10	Adenoviral vector *via* i.n. route	Mice	Induce specific immune response and protection from Mtb challenge	[33, 110, 111]
Ag85B, ESAT-6	Delivered by LTK63 vehicle *via* i.n. route	Mice	Increase anti-Mtb-specific CD4 T cells	[12]
Soluble mycobacterial antigens	Directly delivered by i.n. route	Mice	Restore antigen specific immune responses	[57]
ulticomponent subunits recombinant proteins of Mtb	Delivered with DDA-MPL vehicle *via* i.n. route	Mice	Induce strong antigen-specific T-cell responses	[112]
MPT51	Delivered by Lentiviral vector *via* intratracheal route	Mice	Induce MTP51-specific CD8 T cells and decrease the number of Mtb in lung following challenge	[113]

^+^ MDP: muramyl dipeptide; PLA: poly(lactide); VSV: vesicular stomatitis virus.
